# Development of a Genome-Scale Metabolic Model and Phenome Analysis of the Probiotic *Escherichia coli* Strain Nissle 1917

**DOI:** 10.3390/ijms22042122

**Published:** 2021-02-20

**Authors:** Dohyeon Kim, Youngshin Kim, Sung Ho Yoon

**Affiliations:** Department of Bioscience and Biotechnology, Konkuk University, Seoul 05029, Korea; dhkim92@konkuk.ac.kr (D.K.); kimyssam@konkuk.ac.kr (Y.K.)

**Keywords:** *Escherichia coli* Nissle 1917, probiotics, metabolic network model, phenome analysis, flux balance analysis

## Abstract

*Escherichia coli* Nissle 1917 (EcN) is an intestinal probiotic that is effective for the treatment of intestinal disorders, such as inflammatory bowel disease and ulcerative colitis. EcN is a representative Gram-negative probiotic in biomedical research and is an intensively studied probiotic. However, to date, its genome-wide metabolic network model has not been developed. Here, we developed a comprehensive and highly curated EcN metabolic model, referred to as iDK1463, based on genome comparison and phenome analysis. The model was improved and validated by comparing the simulation results with experimental results from phenotype microarray tests. iDK1463 comprises 1463 genes, 1313 unique metabolites, and 2984 metabolic reactions. Phenome data of EcN were compared with those of *Escherichia coli* intestinal commensal K-12 MG1655. iDK1463 was simulated to identify the genetic determinants responsible for the observed phenotypic differences between EcN and K-12. Further, the model was simulated for gene essentiality analysis and utilization of nutrient sources under anaerobic growth conditions. These analyses provided insights into the metabolic mechanisms by which EcN colonizes and persists in the gut. iDK1463 will contribute to the system-level understanding of the functional capacity of gut microbes and their interactions with microbiota and human hosts, as well as the development of live microbial therapeutics.

## 1. Introduction

The nonpathogenic *Escherichia coli* strain Nissle 1917 (EcN) is among the most studied probiotic bacteria [1,2]. Over 100 years, EcN has been actively used as a pharmaceutical product (trade name: Mutaflor) for the treatment of intestinal disorders, such as inflammatory bowel disease, ulcerative colitis, and diarrhea [2]. EcN is a successful colonizer of the human gut, possessing strong antagonistic activity against entero-pathogens and immunomodulatory properties. The persistent colonization of EcN confers an advantage over Gram-positive probiotic strains, such as lactic acid bacteria, which transiently colonize the gut mucosa [1].

EcN has been genetically engineered to diagnose, prevent, and treat diseases owing to its facile genetics and biosafety profile [3]. Recombinant EcN was constructed to secrete human epidermal growth factors to heal wounds in human intestinal epithelial cells [4]. It was engineered to sense and kill *Pseudomonas aeruginosa* in *Caenorhabditis elegans* and mouse infection models [5]. EcN can selectively colonize and replicate in solid tumors, and its tumor-targeting ability has been exploited to bind to cancer cells and produce cytotoxic compounds in tumor-bearing mice [6,7,8]. EcN was developed as a live microbial therapeutic to treat metabolic disorders, such as phenylketonuria [9] and hyperammonemia [10]. With advances in synthetic and systems biology, EcN is expected to be engineered to develop safer, cheaper, and more effective therapeutics for a wide range of diseases [3].

A genome-scale metabolic network model is a computational framework that allows the prediction of metabolic flux values for a whole set of metabolic reactions using optimization techniques, such as flux balance analysis (FBA) [11,12]. Metabolic models of diverse organisms are used for various purposes, including the prediction of possible functions and phenotypes, understanding genotype–phenotype relationships, and development of metabolically engineered microbes [6]. Moreover, metabolic models of gut microbes can provide insights into the interactions of microbes with each other as well as their host [13]. Strain-specific metabolic models have been reconstructed for nonpathogenic and pathogenic *E. coli* strains [14]. In particular, highly curated and fine-tuned metabolic network models have been developed for the laboratory strains of K-12 MG1655 [15] and BL21(DE3) [16]. Although genome-scale metabolic models were reconstructed for 773 strains of the human gut microbiota [17], the probiotic EcN model has not been developed. Considering the clinical and pharmaceutical importance of EcN, an accurate and comprehensive metabolic model needs to be developed for the systems and synthetic biology of EcN.

In this study, we reconstructed a comprehensive and highly curated metabolic network model of EcN based on comparative genome and phenome analyses. A phenotype microarray (PM) test was performed to validate and update the metabolic model, and the reconstruction was extensively manually curated. Combined with results from the phenome analysis, metabolic simulation using the model identified genetic determinants of EcN-specific phenotypes. Further, genetic and metabolic factors responsible for intestinal colonization of EcN were identified.

## 2. Results

### 2.1. Reconstruction of the Draft Metabolic Model

The EcN genome (RefSeq accession number NZ_CP007799) [18] is poorly annotated; therefore, a draft EcN metabolic model was reconstructed based on homology and the three previously reconstructed *E. coli* metabolic models that are the most highly curated K-12 MG1655 (model id: iML1515) [15] and two of EcN’s closest related *E. coli* strains, uropathogenic CFT073 (ic_1306) [14] and asymptomatic ABU83972 (iECABU_c1320) [14]. Genome annotation [19] and iML1515 [15] of *E. coli* K-12 intestinal commensal strain MG1655 were used as the gold standard for the reconstruction of the EcN metabolic model.

The reconstruction was initiated from the homology searches of the genomes of K-12 MG1655 (NC_000913) [19], CFT073 (NC_004431) [20], and ABU83972 (NC_017631) [21] against the EcN genome (NZ_CP007799) [18]. In total, 1340 metabolic genes were shared by the EcN and K-12 genomes, and 2252 metabolic reactions associated with them were identified in the K-12 metabolic model. A total of 147 metabolic genes and 204 metabolic reactions were shared by EcN and CFT073 but not K-12. There were no metabolic reactions that were shared only in EcN and ABU83972 but not K-12 and CFT073. We added 397 exchange reactions for external metabolites that were not associated with genes.

There were many missing reactions owing to incorrect, missing, or low-quality reactions listed in CFT073 and ABU83972 reconstructions, which were generated through the automatic pipeline [14]. Thus, the MetaCyc database [22] was further explored to identify 13 metabolic reactions that were not included in the metabolic models of K-12, CFT073, and ABU83972 (Supplementary Table S1). Among them, two metabolic reactions existed only in the EcN genome along with their associated genes and metabolites. Three metabolic reactions (DXPRIi, PANTS, and TDSK) were added to the model during manual gap filling. The draft model was further revised based on the PM results (see below).

### 2.2. Phenome Analysis

**Figure 1:**
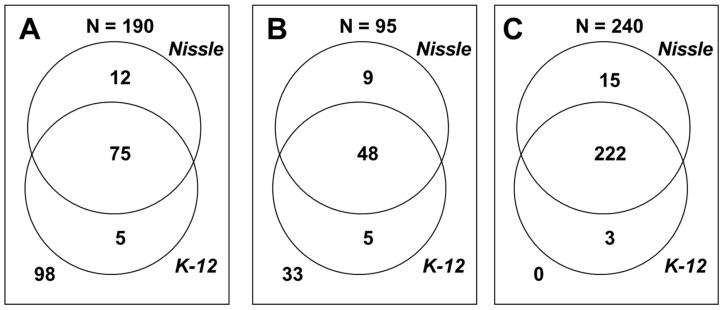
Comparison of phenotype microarray (PM) tests of *E. coli* strains Nissle 1917 and K-12 MG1655. (**A**) Carbon sources (PM1 and PM2). (**B**) Nitrogen sources (PM3). (**C**) Inhibitory compounds, such as antibiotics, antimetabolites, and other inhibitors (PM11 to PM20). The numbers indicate the number of nutrients on which cells grew.

**Figure 2:**
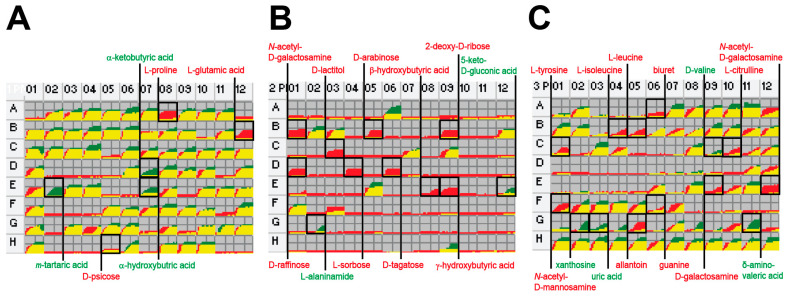
Comparison of carbon and nitrogen source utilization of *E. coli* strains Nissle 1917 and K-12 MG1655. Growth curves in all the cells are shown for *E. coli* Nissle 1917 (red) and K-12 MG1655 (green). (**A**) Carbon sources (PM1). (**B**) Carbon sources (PM2). (**C**) Nitrogen sources (PM3).

We used PM to identify a wide range of digestible and inhibitory substrates through growth behavior [23]. To identify strain-specific characteristics, PM data of EcN were compared to that of K-12, which was previously published by our group [24] (Figure 1, Supplementary Figure S1, and Supplementary Table S2). Among the 190 different carbon sources (PM1 and PM2) and 95 nitrogen sources (PM3), EcN grew aerobically on 87 carbon and 57 nitrogen sources, and K-12 grew on 80 carbon and 53 nitrogen sources. Compared to K-12, only EcN grew on 12 carbon sources (*N*-acetyl-d-galactosamine, D-arabinose, 2-deoxy-d-ribose, L-glutamic acid, β-hydroxy-butyric acid, γ-hydroxy-butyric acid, D-lactitol, L-proline, D-psicose, D-raffinose, L-sorbose, and D-tagatose) and nine nitrogen sources (*N*-acetyl-d-galactosamine, allantoin, biuret, L-citrulline, D-galactosamine, guanine, L-isoleucine, L-leucine, and L-tyrosine), whereas it did not grow on five carbon sources (L-alaninamide, α-hydroxy-butyric acid, α-keto-butyric acid, 5-keto-d-gluconic acid, and *m*-tartaric acid) and five nitrogen sources (δ-amino-*N*-valeric acid, alloxan, uric acid, D-valine, and xanthosine) (Figure 2).

EcN and K-12 grew similarly on phosphorus and sulfur sources (PM4), with the exception of K-12 growth on three phosphorus sources (guanosine-3′,5′-cyclic monophosphate, 2-deoxy-d-glucose-6-phosphate, and cytidine-3′-monophosphate). Both grew on all the cells of PM5 (nutrient supplements), including that of the negative control. EcN grew on the majority of peptide nitrogen sources (PM6–PM8); however, K-12 could not utilize various valine-containing dipeptides owing to the frameshift mutation of *ilvG*, which encodes the subunit of acetohydroxy acid synthase II responsible for valine resistance [24,25]. K-12 was more tolerant to high osmolarity (PM9). Overall, EcN and K-12 showed better cell growth under alkaline (pH 9.5) and acidic conditions (pH 4.5), respectively (PM10).

EcN was more tolerant than K-12 to inhibitory compounds, such as antibiotics, antimetabolites, and other inhibitors (PM11–PM20). Of the 240 different inhibitory compounds tested, EcN and K-12 grew in the presence of 237 and 225 compounds with at least one of four concentrations, respectively. Compared to K-12, only EcN grew in wells containing at least one concentration of 15 compounds (cefotaxime, 5-chloro-7-iodo-8-hydroxyquinoline, erythromycin, 2,2′-dipyridyl, 5-fluorouracil, neomycin, paromomycin, pipemidic acid, potassium tellurite, semicarbazide hydrochloride, sisomicin, sodium selenite, streptomycin, sulfisoxazole, and tobramycin). EcN did not grow in the wells containing three antibiotics (sodium *m*-arsenite, sodium metavanadate, and sodium orthovanadate) at any concentration (Supplementary Table S2).

### 2.3. Gene Clusters Responsible for the Phenotypic Differences

**Figure 3:**
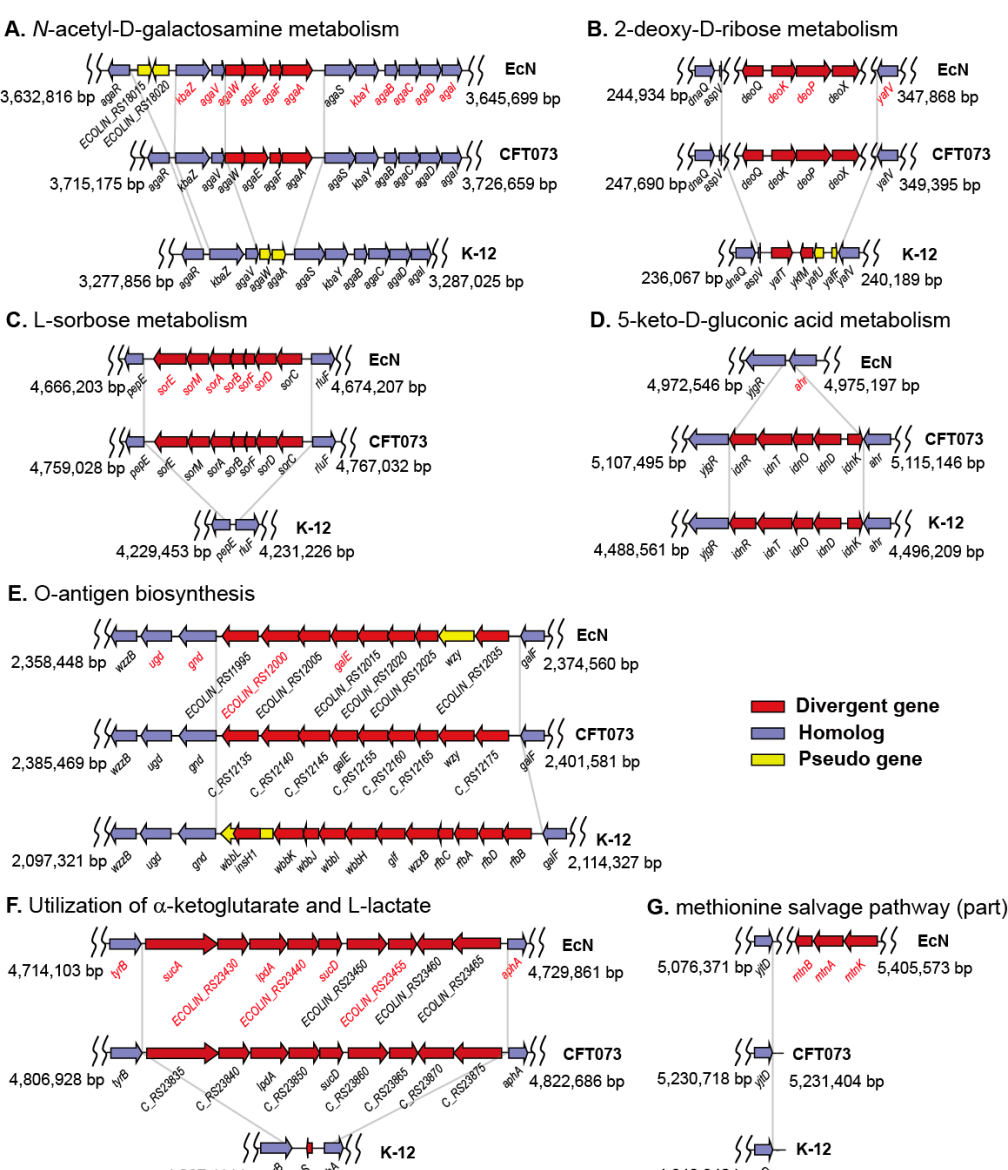
Examples of different metabolic gene clusters in the genomes of *E. coli* strains Nissle 1917, CFT073, and K-12 MG1655. Gene clusters for *N*-acetyl-d-galactosamine metabolism (**A**), 2-deoxy-d-ribose metabolism (**B**), L-sorbose metabolism (**C**), 5-keto-d-gluconic acid metabolism (**D**), O-antigen biosynthesis (**E**), anaerobic utilization of α-ketoglutarate (AKG) and L-lactate (**F**), and a part of methionine salvage pathway (**G**). Gene names in red are for Nissle genes, the reactions of which were included in the Nissle metabolic model.

Many of the phenotypic differences between EcN and K-12 could be correlated with the genotypic differences. Genome comparison of EcN, K-12, and CFT073 identified the genomic regions of EcN that differed from those of K-12, possibly responsible for the phenotypic differences (Figure 3). The related gene clusters of EcN exhibited high levels of structural similarities with those of CFT073; however, they were absent or represented differently in the K-12 genome. The *agaVWEFA* operon for *N*-acetylgalactosamine metabolism [26] is intact in the EcN genome, whereas it is truncated in the K-12 genome (Figure 3A). EcN harbors *deoQKPX* for 2-deoxy-d-ribose utilization [27] (Figure 3B) and *sorEMABFDC* for L-sorbose metabolism [28] (Figure 3C), whereas the K-12 genome does not. Interestingly, EcN does not have *idnK*-*idnDOTR* for a subsidiary pathway for D-gluconate catabolism (GntII), whereas both K-12 and CFT073 do (Figure 3D). However, the PM test showed that EcN effectively grew on D-gluconic acid and 5-keto-d-gluconic acid as the sole carbon source. This agrees with a previous report showing that gluconate catabolism is primarily mediated by the GntI system (encoded by *gntT*, *gntU*, and *gntK*), and that the GntII system is primarily involved in the catabolism of L-idonic acid in which D-gluconate is an intermediate [29].

The comparison of gene clusters for O-antigen biosynthesis revealed that EcN has a nonfunctional *wzy* gene encoding the O-antigen polymerase gene (Figure 3E), which results in the semi-rough O6 lipopolysaccharide (LPS) phenotype and serum sensitivity of EcN [30]. CFT073, which shows smooth LPS expression and serum resistance, has an intact gene cluster for O6 antigen biosynthesis [31]. Rough and serum-sensitive K-12 cannot synthesize the O16 antigen owing to the disruption of the rhamnosyl transferase gene (*wbbL*) by insertion element [32]. Thus, it can be speculated that the difference between EcN and K-12 in O-antigen biosynthetic gene clusters is responsible for the higher resistance of EcN to antibiotics than that of K-12. Gene clusters unrelated to the observed phenotypic differences were also inspected. In contrast to K-12, EcN has gene clusters associated with the anaerobic utilization of α-ketoglutarate (AKG) and L-lactate (Figure 3F) and a part of the methionine salvage pathway (MSP) [33] (Figure 3G).

### 2.4. Revision and Validation of the Metabolic Model

The draft reconstruction was updated by comparing the simulated growths from the FBA and experimental growths from the PM test under aerobic growth conditions. For 190 types of carbon sources (PM1 and PM2) and 95 nitrogen sources (PM3), differences in the simulated and experimental growths were identified in the utilization of 25 carbon and 19 nitrogen sources (Supplementary Table S3), which corresponded to 70.9% of the model accuracy.

The incongruences in the simulated and experimental growths were reflected in the modification of the model (Table 1). False negatives (growth from the PM test and non-growth from simulation) were observed for *N*-acetyl-d-glucosamine, *N*-acetyl-β-d-mannosamine, fructose, sorbitol, mannitol, mannose, and glucosamine, and these are carbohydrates transported by the sugar phosphotransferase system (PTS). Among the genes associated with PTS, we found that *ptsI* encoding PTS enzyme I was frameshifted in the EcN genome (NZ_CP007799). As PtsI activity is essential for *E. coli* growth on these PTS sugars [34], we sequenced the *ptsI* gene, and its DNA sequence was found to be intact and the same as that of *E. coli* ABU83972. Thus, we added transport reactions for these PTS sugars. We added one transport reaction and two intracellular reactions associated with the *sorEMABFDC* operon genes associated with L-sorbose metabolism. We added 13 dipeptidase reactions (catalyzed by PepD aminopeptidase) to break down 13 dipeptides and one dipeptidase reaction (catalyzed by PepQ prolidase) specifically hydrolyzing dipeptides containing a proline residue at the carboxy-terminal end [35] (Supplementary Table S1). We further added one exchange reaction and three transport reactions for the utilization of each of the 14 dipeptides as carbon or nitrogen sources. For example, glycyl-L-glutamic acid was utilized by adding EX_gly_glu__L_e, GLYLGLUtex, GLYLGLUtpp, GLYLGLUabcpp, and AMPEP8.

False positives (growth from simulation and non-growth from the PM test) were observed for the utilization of 12 substrates (acetoacetic acid, γ-amino-N-butyric acid, butyric acid, D-cellobiose, ethanolamine, inosine, L-ornithine, 1,2-propanediol, D-tartaric acid, L-tartaric acid, uric acid, and xanthine) as the sole carbon or nitrogen source. To match the predictions and experimental results, six reactions were removed from the draft model, as no relevant genes were retrieved in the MetaCyc [22] and/or KEGG [36] databases.

Eight reactions were reported to be active only under anaerobic (DTARTD, LCARS [37], SUCTARTtpp [38], TARTD, TARTRt7pp [39]) and stressful conditions (ARGAGMt7pp [40] and CELLBpts [41]); they were deactivated by constraining their flux to zero in the FBA simulation under aerobic conditions (Table 1). PTRCORNt7pp (for putrescine/ornithine antiporter) was deactivated because its associated gene (*potE*) was reported to show weak gene expression levels under normal culture conditions [42].

EcN has fully functional pathways for central carbon metabolism. It is also fully functional in the de novo syntheses of all essential nucleotides, amino acids, fatty acids, and LPS. The growth kinetics of EcN using diverse carbon sources was comparable to that of K-12 (Supplementary Figure S1), demonstrating full activity in aerobic respiration. During the model validation, the *aceF* gene, which was annotated to be frameshifted in the EcN genome (NZ_CP007799), was sequenced to be identical to that in the CFT073 genome. The *aceF* encodes the subunit of pyruvate dehydrogenase (PDH), catalyzing a preparatory step to the tricarboxylic acid (TCA) cycle by converting pyruvate from glycolysis into acetyl-CoA during aerobic respiration, and thus, it should be intact to explain the observed rapid growth of EcN under aerobic conditions.

All the entities of the metabolic model, such as metabolic reactions, genes, metabolites, and gene–protein–reaction rules, were manually curated. The final EcN metabolic model, referred to as iDK1463, comprised 1463 genes, 1313 unique metabolites, and 2984 metabolic reactions (Table 2). The simulated growth qualitatively agreed with the PM results for 240 of 190 carbon and 95 nitrogen sources, and the predictive accuracy of our model can be considered to be 84.2%. The quality and scope of iDK1463 were further evaluated using a metabolic model-testing suit MEMOTE [43], achieving an overall score of 89.4%, which is close to that of the K-12 model iML1515 (90.6%) (Supplementary Figure S2). The model can be downloaded in Excel (Supplementary Table S4) and SBML formats (Supplementary File), which are input to FBA software [44,45].

### 2.5. Gene Essentiality Analysis

FBA was performed to predict the reactions essential for cell growth of EcN and K-12 strains under aerobic conditions using glucose as the sole carbon source. A total of 195 genes were predicted to be essential for each EcN and K-12, of which 191 genes were shared. The predictions were compared with 249 essential genes determined by experimental genome-wide gene-knockout screens performed for K-12 under glucose growth conditions [15]. The predictions using EcN iDK1463 were consistent with the experimental results for 176 genes (accuracy of 70.7%), which is close to that of the K-12 model iML1515 (71.5%). EcN harbored four essential genes (*argI*, *can*, *leuB*, and *purN*) that were not predicted to be essential in K-12, and two of them (*can* and *leuB*) were experimentally validated. Ornithine carbamoyltransferase is involved in the L-arginine biosynthetic pathway, which is encoded by two genes in K-12 (*argF* and *argI*) and one gene in EcN (*argI*) [46]. Carbonic anhydrase is encoded by two genes in K-12 (*can* and *cynT*) and one gene in EcN (*can*) [47]. 3-isopropylmalate dehydrogenase is involved in the L-leucine biosynthetic pathway, which is encoded by two genes in K-12 (*leuB* and *dmlA*) and one gene in EcN (*leuB*) [48]. Phosphoribosylglycinamide formyltransferase, involved in de novo purine biosynthesis, is encoded by two genes in K-12 (*purN* and *purT*) and one gene in EcN (*purN*). A single deletion of *purN* in K-12 did not lead to purine auxotrophy [49]; however, only mutants defective in both *purN* and *purT* resulted in purine auxotrophy [50].

Of the 69 metabolic genes involved in the central catabolic pathways (glycolysis, TCA cycle, and pentose phosphate cycle), nine were reported to be essential for K-12 aerobically growing on glucose [15]. Of the nine essential genes, two genes involved in the TCA cycle (*gltA* encoding citrate synthase and *icd* encoding isocitrate dehydrogenase) were predicted to be essential from FBA using iML1515 of K-12 [15], and this was the same result obtained when iDK1463 was used. The remaining seven genes (*lpd*, *gapA*, *eno*, *fbaA*, *pgk*, *pfkA*, and *tpiA*) represent false positives (i.e., predicted growth while experimental non-growth). False growth prediction can be used to refine the model when more biochemical information is available [12].

### 2.6. Simulation of Utilization of Carbon Sources under Anaerobic Intestinal Environments

iDK1463 was simulated for the anaerobic utilization of the six major sugars in gastrointestinal mucus (*N*-acetylgalactosamine, *N*-acetylglucosamine, *N*-acetylneuraminic acid, L-fucose, D-galactose, and D-gluconate) [51,52,53], four substrates associated with the EcN-specific presence of metabolic gene clusters compared to K-12 (2-deoxy-d-ribose, L-lactic acid, L-sorbose, and AKG), and D-glucose (Figure 3). Glycerol was also tested because it was reported to be a major carbon source of energy supply for intracellularly replicating *Listeria monocytogenes* [54]. From the study on the sugar utilization of EcN in the mouse intestine [55], five of the 12 simulated carbon sources (*N*-acetylneuraminic acid, *N*-acetylgalactosamine, D-galactose, D-gluconate, and L-fucose) supported EcN colonization, while *N*-acetylglucosamine and D-ribose were unnecessary for the intestinal colonization. For FBA under anaerobic conditions, the upper limits of oxygen and each of the carbon sources were set to zero and 20 mmol gDCW^−1^ h^−1^, respectively. Fluxes of six reactions that were deactivated for aerobic FBAs (DTARTD, LCARS, SUCTARTtpp, TARTD, TARTRt7pp, and ARGAGMT7pp) (Table 1) were unconstrained. We further unconstrained the flux mediated by the formate–hydrogen lyase complex, which is oxygen sensitive and is responsible for H_2_ production via formate oxidation under fermentative conditions [56].

**Figure 4:**
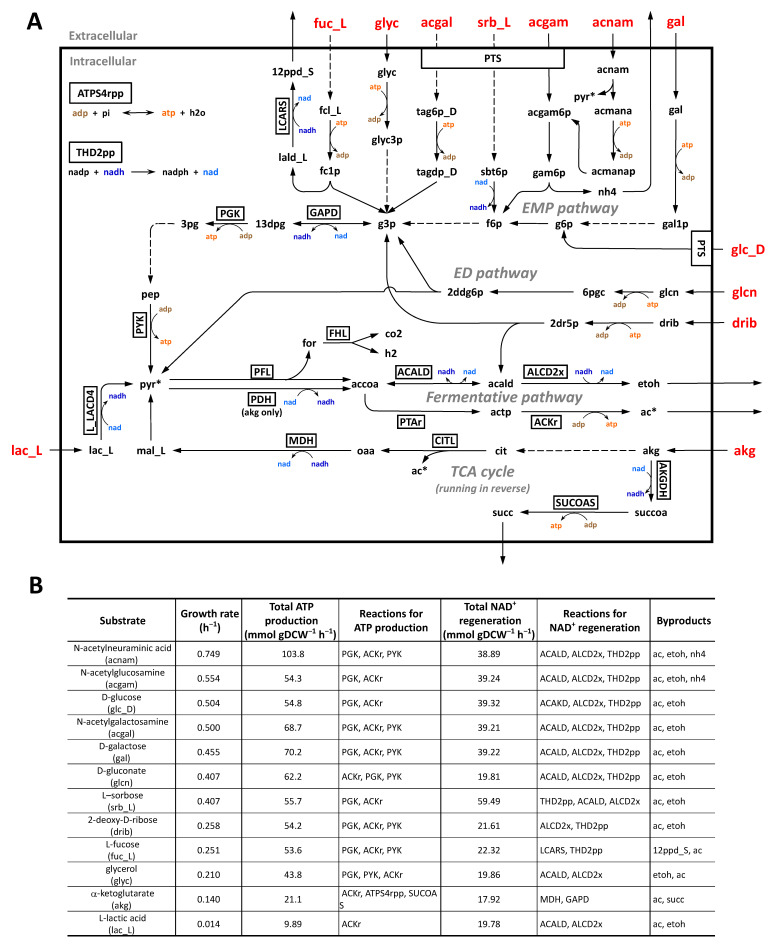
Model predictions of growth capability in the nutrient sources under anaerobic conditions. (**A**) Schematics of the predicted flux distributions. The nine carbon sources simulated are in red. Reactions are in the box. Arrows denote directions of the predicted metabolic fluxes, and dashed arrows indicate the multi-step reaction. Metabolites colored above arrows indicate cofactors (ADP, ATP, NAD, and NADH). Some metabolites (pyruvate and acetate) are duplicated on the map for clearer visualization and are labeled with an asterisk (*). (**B**) Summary of the simulation for the utilization of nine carbon sources. “Reactions for ATP production” and “Reactions for NAD^+^ regeneration” are reactions responsible for >95% of total ATP production and total NAD^+^ regeneration, respectively, in descending order. Abbreviations are given in Supplementary Table S4. For flux balance analysis (FBA) under anaerobic conditions, the upper limits of oxygen and each of the carbon sources were set to zero and 20 mmol gDCW^−1^ h^−1^, respectively.

The simulated flux distribution patterns were depicted for the 12 carbon sources that were fed into the fermentative pathway (Figure 4A). Entry points into the Embden–Meyerhof–Parnas (EMP) pathway were glucose 6-phosphate (from the degradation of D-galactose and D-glucose), fructose 6-phosphate (from *N*-acetylglucosamine, *N*-acetylneuraminic acid, and L-sorbose), and D-glyceraldehyde 3-phosphate (from L-fucose, *N*-acetylgalactosamine, 2-deoxy-d-ribose, and glycerol). D-gluconate was metabolized to D-glyceraldehyde 3-phosphate and pyruvate almost exclusively via the Entner–Doudoroff (ED) pathway. L-lactic acid and AKG were subsequently converted to pyruvate. The predicted growth yield, ATP production, and NAD^+^ regeneration according to the carbon sources are summarized in Figure 4B. The simulated growth rate was highest (0.749 h^−1^) when *N*-acetylneuraminic acid was used as the sole carbon source, followed by *N*-acetylglucosamine (0.554) > D-glucose (0.504) ≈ *N*-acetylgalactosamine (0.5) > D-galactose (0.455) > D-gluconate (0.407) = L-sorbose (0.407) > 2-deoxy-d-ribose (0.258) ≈ L-fucose (0.251) > glycerol (0.210) > AKG (0.14) > L-lactic acid (0.014) (Figure 4). The utilization of one *N*-acetylneuraminic acid molecule led to the formation of two pyruvates from glycolysis and one additional pyruvate catalyzed by *N*-acetylneuraminate lyase (Figure 4A), and thus, the highest amount of ATP was produced via the glycolytic and fermentative pathways.

The catabolism of AKG and L-lactic acid did not include glycolysis, thereby yielding the lowest growth rate. Intracellular AKG was converted to citrate by running the TCA cycle in reverse, then to oxaloacetate and acetate by citrate lyase of an anaerobic enzyme [57]. Oxaloacetate was reduced to malate, allowing NAD^+^ regeneration by malate dehydrogenase (MDH). Malate was converted to pyruvate as a part of gluconeogenesis. Pyruvate was converted to acetyl-CoA with concomitant generation of NADH by PDH, whereas the catabolism of the other eight carbon sources employed anaerobically induced pyruvate formate lyase (PFL), which catalyzes the non-oxidative cleavage of pyruvate to acetyl-CoA and formate [58]. Intracellular L-lactic acid was directly converted to pyruvate with NADH generation by L-lactate dehydrogenase (L-LDH). The prepared pyruvate entered the fermentative pathway by which only acetate was produced for the catabolism of AKG, whereas acetate and ethanol were produced for the L-lactic acid catabolism. The inefficient NAD^+^ regeneration resulted in the lowest ATP production for FBA using L-lactate as the sole carbon source.

## 3. Discussion

Differences in *E. coli* strains of Nissle and K-12 in the utilization of diverse nutrient substrates were identified (Figure 2) and could, in many cases, be correlated with genotypic differences (Figure 3). In general, genome sequence data alone do not allow the identification of the genetic determinants of a given phenotypic variation, and the number of genetic differences even between closely related strains is often too large to be evaluated by gene deletion and complementation tests [59]. The metabolic simulations for nutrient utilization described in this study outline a general strategy for identifying the genetic basis of phenotypic divergence based on clues from comparative genome and phenome analyses. Experimental evidence for the phenotypic differences between the two organisms was provided by the PM tests, and the genetic bases responsible for the phenotypic differences were corroborated by metabolic simulation. Previously, we used this approach to reveal the genotype–phenotype associations between closely related *E. coli* strains B and K-12 [16,24]. Our approach forms a general strategy to understand the genetic basis of phenotypic functions of naturally occurring strains and laboratory-evolved strains.

The spectrum of nutrient utilization and antibiotic resistance is the first step toward a comprehensive understanding of the mechanisms by which commensals and pathogens colonize and persist in the gut in both normal and inflammatory conditions. This is in line with the previous finding that the human commensal *E. coli* K-12 MG1655 and enterohemorrhagic *E. coli* EDL933 grew primarily as single cells dispersed in the mouse cecal mucus layer [60]. In this regard, PM data in this study serve as a compendium of nearly 2000 monoculture phenotypes of EcN over a wide range of digestible substrates and inhibitory substrates.

The phenotypic profiles in this study provide insights into the colonizing capabilities of EcN. Nutrient sources supporting cell growth are important for *E. coli* to colonize the intestine [53]. EcN can use more diverse carbon (87 vs. 80) and nitrogen (57 vs. 53) sources than K-12 (Figure 1). EcN can aerobically grow on 13 sugars (fructose, fucose, galacturonate, gluconate, glucose, glucuronate, lactose, maltose, mannose, *N*-acetyl-d-glucosamine, *N*-acetylneuraminate, ribose, and xylose) and two carboxylic acids (acetate and malate), which were reported to be present in the gastrointestinal tract [52,53]. Commensal *E. coli* strains grow predominantly in the mucus layer [61]. They cannot degrade mucus polysaccharides and rely on other anaerobes for the release of metabolizable sugars. Thus, metabolic interactions with anaerobes and utilization of mucus-derived sugars is important for intestinal colonization by EcN [61,62]. Of the 15 nutrients, K-12 could not grow on the mucus-derived amino sugar of *N*-acetyl-d-galactosamine. Furthermore, it could not grow on 2-deoxy-d-ribose, which promotes pathogenic *E. coli* strains during host colonization because deoxyribose is derived from DNA degradation and is abundant in the intestine [63].

While EcN and K-12 showed high similarity in aerobic respiration, they were different in the anaerobic fermentative pathway. Remarkably, EcN has a large gene cluster for L-lactate biosynthesis, anaerobic utilization of AKG, and their two-component regulatory system (Figure 3F). To date, the genomic island is known to be specifically present in uropathogenic *E. coli* and absent from commensal *E. coli* and intestinal pathogenic *E. coli* [64]. As AKG is abundant in renal proximal tubule cells [65] and activity of AKG dehydrogenase shared by all *E. coli* strains is repressed under anaerobic conditions [66], the anaerobic utilization of AKG by AKG dehydrogenase encoded on the genomic island might be important for the colonization of the urinary tract by uropathogenic *E. coli* [64]. The intestine is supposed to be a major consumer of glutamine, which is the most abundant amino acid in the body and diet [67], and AKG is believed to be the most preferable glutamine derivative [68]. In addition to the quinone-dependent L-LDH (LldD, EC 1.1.2.3) shared by EcN and K-12, EcN has an additional NADH-dependent L-LDH (encoded by ECOLIN_RS23455, EC 1.1.1.27) on the genomic island. L-LDH catalyzes the oxidation of L-lactate to pyruvate, which has been reported to be important for the intracellular growth of *Mycobacterium tuberculosis* in human macrophages [69]. Furthermore, L-lactate is supposed to be a universal carbon source for intracellularly growing bacteria [69], and LldD expression is repressed under anaerobic conditions [70]. Our metabolic simulation demonstrates the anaerobic utilization of AKG and L-lactate without glycolysis (Figure 4). Taken together, the presence of the genomic island seems to be distinctly advantageous to EcN for intestinal colonization through the anaerobic utilization of AKG and L-lactate.

The ED pathway is important for *E. coli* to colonize the mouse intestine as it is the primary route for the catabolism of gluconate and other sugar acids which are available in the mucus layer [71]. Especially, gluconate was found to be a major carbon source for *E. coli* MG1655 to colonize in the mouse intestine [53]. In metabolic simulations of the anaerobic utilization of gluconate (Figure 4), gluconate was predicted to be metabolized via the ED pathway by 97.4% and 77.5%, respectively, in EcN and K-12. This contrasts with the simulated aerobic utilization of gluconate, which was metabolized exclusively via the pentose phosphate pathway to enter the upper part (fructose 6-phosphate and D-glyceraldehyde 3-phosphate) of the EMP pathway in both EcN and K-12. The high contribution of the ED pathway to gluconate metabolism in anaerobic conditions may imply that the ED pathway serves as an important energy-generating pathway for EcN growth in intestinal habitats [71].

Owing to the incomplete MSP, *E. coli* strains cannot synthesize methionine from 5-methylthioribose, which is exported into the culture media as waste [40]. Interestingly, EcN has an operon for the conversion from methylthioribose (MTR) to methylthioribulose-1-phosphate (MTRu-1-P) to 2,3-diketo-5-methylthiopentyl-1-phosphate as in extra-intestinal pathogenic *E. coli* S88 (O45:K1:H7) [33] (Figure 3G). However, no genes encoding downstream enzymes of MSP were found in EcN [72]. MTRu-1-P is highly reactive; therefore, interrupting MSP at the point where MTRu-1-P is produced appears to be metabolically impractical, and thus, the presence of an unknown alternative pathway for metabolizing MTRu-1-P has been suggested [33,72].

The growth of intracellular pathogens relies on host metabolites and depends on the efficient usage of the nutrients at various spatial locations and stages of intracellular growth [61]. The bipartite metabolism, a network topology with separate nutrient usage subnetworks for specific catabolic and anabolic purposes, is often found in many intracellular pathogens, such as *Coxiella burnetii*, *Chlamydia trachomatis*, *Legionella pneumophila*, and *Listeria monocytogenes* [73]. Little is known about the features of the complex metabolic and regulatory interactions between intracellular pathogens. Although nutrient-specific control over central metabolism by a carbon storage regulator (Csr) has been reported for EcN [74], studies on the bipartite metabolism of EcN are yet to be conducted. Once knowledge of the specific regulatory events and nutrient composition of intestinal habitats for EcN growth is accumulated, EcN iDK1463 can be utilized to elucidate potential bipartite metabolism by tracking carbon fluxes derived from various substrates, as exemplified in Figure 4.

In general, the most common probiotics are Gram-positive strains, such as *Lactobacillus* and *Bifidobacterium* species [75]. EcN is a representative Gram-negative probiotic and a model microorganism in biomedical research [76]. EcN has also been extensively engineered for therapeutic applications [3]. In the very early stages of systems and synthetic biology of EcN, iDK1463 can act as a framework for system modeling and integration of various omics data. This would facilitate the rational design of efficient probiotic interventions. Furthermore, iDK1463 can be applied to understand bacteria–bacteria as well as bacteria–host interactions occurring in the intestinal ecosystem.

## 4. Materials and Methods

### 4.1. Identification of Metabolic Genes and Reactions

The complete genome sequence of EcN (RefSeq accession number: NZ_CP007799) [18] was used for the functional annotation of genes based on homology searches. For each of the genomes of *E. coli* strains of K-12 MG1655 (NC_000913) [19], CFT073 (NC_004431) [20], and ABU83972 (NC_017631) [21], the protein sequences were BLASTP-searched against whole protein sequences of the EcN genome, using BLOSUM62 as a scoring matrix. If the percentage of conserved substitutions (percent positives) was over 90% and the aligned region was both over 90% of the lengths of the query and the hit, the pair of sequences was considered a homolog. The EDGAR server (version 2.3) [77] was further employed to find homologs in the EcN genome, with default settings.

Genes with potential metabolic roles were identified from the list of genes contained in the metabolic models of K-12 MG1655 (model id: iML1515), CFT073 (ic_1306), and ABU83972 (iECABU_c1320), which were retrieved from the BiGG models database [78], and their associated enzymes and biochemical reactions were also retrieved. Metabolic genes with no homologs in K-12, CFT073, and ABU83972 were queried to MetaCyc [22] to find metabolic reactions, using the MATLAB toolbox RAVEN version 2.0 [79]. The resulting gene–protein–reaction rules were manually revised.

### 4.2. PM Test

*E. coli* strain Nissle 1917 (Mutaflor, DSM 6601, serotype O6:K5:H1) was provided by Ardeypharm GmbH, (Herdecke, Germany). PM tests were performed as previously described [16]. The PM plates (Biolog Inc., Hayward, CA, USA) comprised 20 96-well plates containing different sources of carbon (PM1 and PM2), nitrogen (PM3), phosphorus, and sulfur (PM4); auxotrophic supplements (PM5); peptide nitrogen sources (PM6–PM8); and osmolytes (PM9). The PM10 plate was used to monitor cell growth on the pH stress, and PM11 to PM20 plates contained inhibitory compounds, such as antibiotics and antimetabolites. PM data of *E. coli* K-12 MG1655, which were purchased from American Type Culture Collection (ATCC), were retrieved from our previous study [24].

Cells were grown overnight at 37 °C on a Biolog universal growth (BUG) + B agar plate. Colonies were picked from the agar surface and suspended in inoculating fluid (IF) containing the indicator dye tetrazolium violet. IF-0 media were used for plates PM1 to PM8 and IF-10 for plates PM9 to PM20. Sodium succinate was added with ferric citrate to the inoculation solution of plates PM3 to PM8. All PM plates were inoculated with cell suspensions at 100 μL/well and incubated in an OmniLog incubator (Biolog Inc., Hayward, CA, USA) at 37 °C for 48 h. Four independent PM tests were performed using sodium succinate as the sole carbon source for the plates PM3 to PM8. The PM data were analyzed using the R package opm [80].

### 4.3. FBA

FBA was performed using the COBRApy software [45]. The maximum growth rate derived from the core biomass equation of K-12 iML1515 was set as the default objective function. Previously reported values of 59.8 and 8.4 mmol gDCW^−1^ h^−1^ of EcN were used as growth-associated maintenance energy (GAM) and non-growth-associated maintenance energy (NGAM), respectively [74]. The upper limits of glucose and oxygen were set to 10 and 18.5 mmol gDCW^−1^ h^−1^, respectively, unless otherwise mentioned.

For simulation of cell growth on PM plates for testing utilization of carbon (PM1 and PM2) and nitrogen (PM3) sources, the composition of defined media used in the PM plates was retrieved from BioCyc [22], and the same medium composition was used as the maximum uptake rate of the corresponding component. For simulations on PM1 and PM2, the maximum uptake rate of each carbon source was set to 10 mmol gDCW^−1^ h^−1^. For PM3 simulations, the maximum uptake rates of succinate and a nitrogen source were set to 10 and 5 mmol gDCW^−1^ h^−1^, respectively. A substrate was considered to be not utilized if the growth rate was <5% of the growth objective value calculated for the wild-type strain.

To identify the reactions that are essential for cell growth, the maximum growth rate was simulated when each reaction was removed from the reconstructed metabolic model using COBRApy [45]. An unrestrained supply of all the substrates was assumed. A reaction was considered essential if its removal from the model reduced the growth rate below the default threshold of 5% of the growth objective value calculated for the wild-type strain.

### 4.4. DNA Sequencing

For DNA sequencing of the *ptsI* and *aceF* genes, three sets of primers were prepared for each gene divided into three parts (Supplementary Table S5). Considering that the quality score of sequencing result was low in the front and back of the sequences, approximately 200 bp was left as a margin at the front and rear of the gene sequence, and each part was overlapped by approximately 300 bp. The gene was amplified using a PCR T100 thermal cycler (BioRad, Hercules, CA, USA) with genomic DNA of EcN as a template. The PCR product was purified using Expin PCR SV (GeneAll Biotechnology, Seoul, Korea). Sanger sequencing was performed using the BigDye Terminator v3.1 Cycle Sequencing Kit (Applied Biosystems, Thermo Fisher Scientific Inc., Waltham, MA, USA). The sequencing reaction was run using a DNA Engine Tetrad 2 Peltier Thermal Cycler (BioRad, Hercules, CA, USA), and each of the three parts was sequenced using three different forward primers. After removal of unincorporated dNTPs and other ingredients, the final products were loaded onto an ABI 3730xl DNA Analyzer (Applied Biosystems, Thermo Fisher Scientific Inc., Waltham, MA, USA) to obtain the sequencing results.

### 4.5. Availability of Data and Materials (Nucleotide Sequence Accession Numbers)

The sequences of *ptsI* and *aceF* of *E. coli* Nissle 1917 were deposited in GenBank under accession numbers MW239125 and MW239126, respectively.

## Figures and Tables

**Table 1:** Model refinement based on comparison of the simulated and experimental growths.

Substrates	PM ^a^	Prediction ^b^ (Draft/iDK1463)	Associated Reactions ^c^	Modification
**Carbon Sources**				
acetoacetic acid	-	+/−	ACACtex	Deletion
γ-amino-N-butyric acid	-	+/−	ABUTtex	Deletion
butyric acid	-	+/−	BUTt2rpp	Deletion
D-cellobiose	-	+/−	CELLBpts	Deactivated
1,2-propanediol	-	+/−	LCARS	Deactivated
L-ornithine	-	+/−	PTRCORNt7pp	Deactivated
L-sorbose	+	−/+	EX_srb__L_e, SRBtex, SRBptspp, SRB1PR	Addition
PTS sugars ^d^	+	−/+	ACGAptspp, ACMANAptspp, FRUptspp, SBTptspp, MNLptspp, MANptspp, GAMptspp	Addition
D-tartaric acid	-	+/−	DTARTD, SUCTARTtpp	Deactivated
L-tartaric acid	-	+/−	TARTD, TARTRt7pp	Deactivated
**Nitrogen Sources**				
ethanolamine	-	+/−	ETHAtex	Deletion
inosine	-	+/−	URIC	Deletion
uric acid	-	+/−	URIC	Deletion
xanthine	-	+/−	XANtex	Deletion

The complete list of experimental and simulated cell growth is available in Supplementary Table S3. ^a^ Cell growth from PM tests is categorized into non-growth (“−”) or growth (“+”) based on statistical analysis of four growth replicates of each strain (see Supplementary Table S2 for details). ^b^ Simulated cell growth on each carbon and nitrogen source by flux balance analysis: non-growth (“−”), growth (“+”). ^c^ Detailed information for the reactions is listed in Supplementary Table S4. ^d^ Carbohydrates transported by sugar phosphotransferase system (PTS) (*N*-acetyl-d-glucosamine, *N*-acetyl-β-d-mannosamine, D-fructose, D-glucosamine, D-mannitol, D-mannose, and D-sorbitol).

**Table 2:** Components of the metabolic network model of *E. coli* Nissle 1917.

Metabolic Model	iDK1463
Genes	1463
Metabolic reactions	2984
Enzymatic reactions	1633
Transport reactions	903
Exchange reactions	441
Demand reactions	7
Gene-reaction association	2986
Gene-associated reactions	2368
Not gene-associated reactions	578
Spontaneous reactions	40
Metabolites	2112
Cytoplasmic	1160
Periplasmic	509
Extracellular	443
Unique metabolites	1313

## Data Availability

The data presented in this study are available in supplementary materials.

## Supplementary Materials

The following are available online at https://www.mdpi.com/1422-0067/22/4/2122/s1.

## Abbreviations

AKG	α-ketoglutarate
EcN	*Escherichia coli* Nissle 1917
ED	Entner–Doudoroff
EMP	Embden–Meyerhof–Parnas
FBA	Flux balance analysis
L-LDH	L-lactate dehydrogenase
LPS	Lipopolysaccharide
MSP	Methionine salvage pathway
PM	Phenotype microarray
PTS	Phosphotransferase system
