# Supplementary material for: Development of a Genome-Scale Metabolic Model and Phenome Analysis of the Probiotic Escherichia coli Strain Nissle 1917
Source: Int J Mol Sci. 2021 Feb 20;22(4):2122. doi: 10.3390/ijms22042122 (PMC7924626; doi:10.3390/ijms22042122)
Supplement: Supplementary file 1 [file ijms-22-02122-s001.zip › Figure S1, Figure S2, Table S5.docx]

**
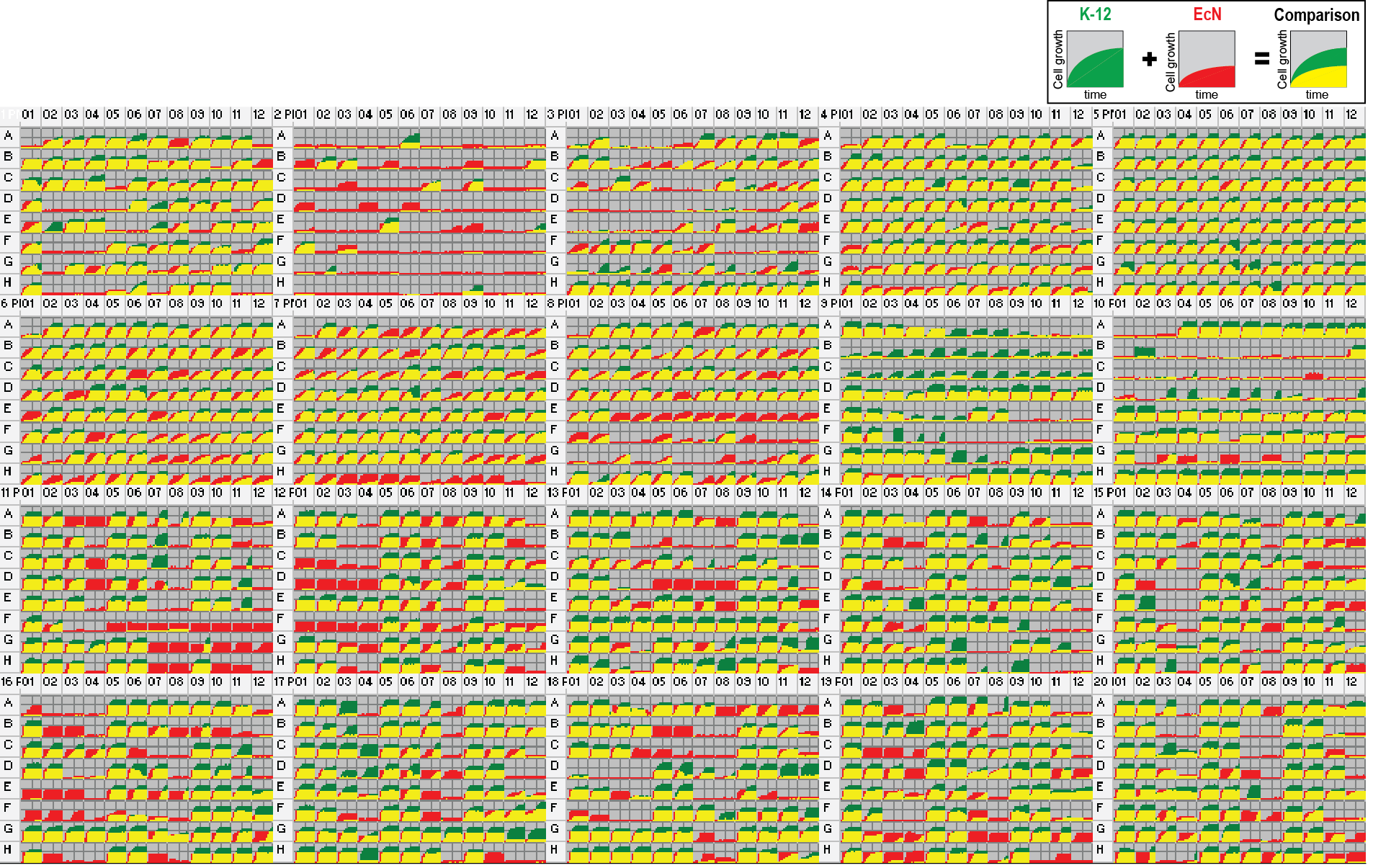
**

**Figure S1.** Comparison of phenotype microarrays (PMs) of *E. coli* strains Nissle 1917 and K-12 MG1655. Growth curves during 48 h are colored yellow for similar growth of Nissle and K-12, red for faster growth of Nissle, and green for faster growth of K-12. The PM plates (Biolog Inc.) comprise 20 96-well microplates containing different sources of carbon (PM1 and PM2), nitrogen (PM3), phosphorus, and sulfur (PM4), auxotrophic supplements (PM5), peptide nitrogen sources (PM6–PM8), osmolytes (PM9), pH stress (PM10), and inhibitory compounds such as antibiotics, antimetabolites, and other inhibitors (PM11–PM20). Sodium succinate was supplemented as the main carbon source (PM3–PM8). Detailed information on PM tests can be found at www.biolog.com.


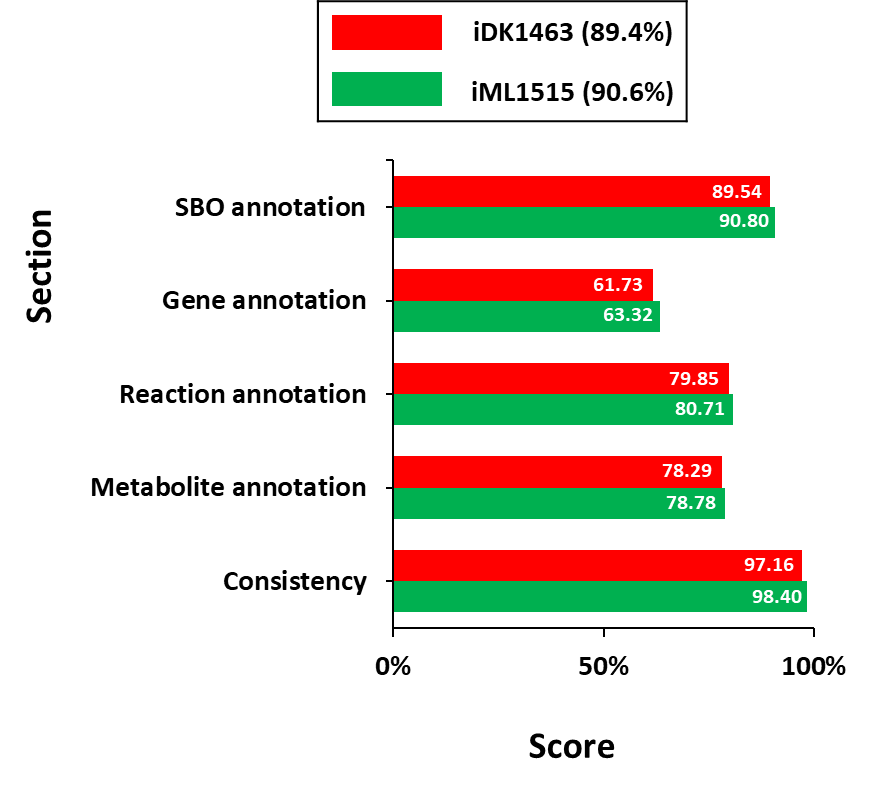


**Figure S2.** Comparison of scores provided by MEMOTE (for metabolic model tests). “Overall score” is denoted in the legend.

**Table S5.** Primers used for DNA sequencing of *ptsI* and *aceF.*

| **Primer** | **Oligonucleotide Sequence (5′→3′)** |
| --- | --- |
| ptsI1-F | CAGCGCGAAAAGCCTGTTTA |
| ptsI1-R | GACCGTCCAGCGTAATAGC |
| ptsI2-F | AACCGCACAGCTGAACCTGAA |
| ptsI2-R | AGTTCAGCACGGACGGTGAC |
| ptsI3-F | GTGCCTCTGCTTTCGGTAAA |
| ptsI3-R | TCAACGCCGCTATCAGATTCG |
| aceF1-F | ACCTGCGTCACCACTTTGAA |
| aceF1-R | TTCAGTTCCTTCACGACGCC |
| aceF2-F | TTTGCTGGCACCGTGAAAGA |
| aceF2-R | ACTGTTGAAGCGAGGCATCT |
| aceF3-F | GCGAAGACGTTCAGGCTTAC |
| aceF3-R | CGGGTCGTTCTATCCGTCG |
